# Obesity-Related Oxidative Stress: the Impact of Physical Activity and Diet Manipulation

**DOI:** 10.1186/s40798-015-0031-y

**Published:** 2015-09-23

**Authors:** Chun-Jung Huang, Matthew J. McAllister, Aaron L. Slusher, Heather E. Webb, J. Thomas Mock, Edmund O. Acevedo

**Affiliations:** 1 Exercise Biochemistry Laboratory, Department of Exercise Science and Health Promotion, Florida Atlantic University, 777 Glades Road, FH11A-126B, Boca Raton, FL 33431 USA; 2 Department of Kinesiology, Mississippi State University, Starkville, MS USA; 3 Department of Kinesiology and Health Sciences, Virginia Commonwealth University, Richmond, VA USA; 4 Department of Kinesiology, Texas A&M University–Corpus Christi, Corpus Christi, TX USA

## Abstract

Obesity-related oxidative stress, the imbalance between pro-oxidants and antioxidants (e.g., nitric oxide), has been linked to metabolic and cardiovascular disease, including endothelial dysfunction and atherosclerosis. Reactive oxygen species (ROS) are essential for physiological functions including gene expression, cellular growth, infection defense, and modulating endothelial function. However, elevated ROS and/or diminished antioxidant capacity leading to oxidative stress can lead to dysfunction. Physical activity also results in an acute state of oxidative stress. However, it is likely that chronic physical activity provides a stimulus for favorable oxidative adaptations and enhanced physiological performance and physical health, although distinct responses between aerobic and anaerobic activities warrant further investigation. Studies support the benefits of dietary modification as well as exercise interventions in alleviating oxidative stress susceptibility. Since obese individuals tend to demonstrate elevated markers of oxidative stress, the implications for this population are significant. Therefore, in this review our aim is to discuss (i) the role of oxidative stress and inflammation as associated with obesity-related diseases, (ii) the potential concerns and benefits of exercise-mediated oxidative stress, and (iii) the advantageous role of dietary modification, including acute or chronic caloric restriction and vitamin D supplementation.

## Key Points

- Acute exercise is a small source of oxidative stress, while chronic exercise elicits protective adaptations against oxidative damage.
- Chronic ingestion of energy-rich foods may contribute to obesity, while acute ingestion may also elicit potentially adverse metabolic responses including oxidative stress.
- Caloric restriction may attenuate oxidative stress and serve as a beneficial weight loss intervention for obese individuals.

## Review

### Introduction

The prevalence of obesity continues to increase in the USA, with recent reports indicating over 64.1 % of American women and 72.3 % of American men are categorized as overweight and/or obese [body mass index (BMI) ≥ 25 kg/m^2^] [1]. Obese individuals have demonstrated markers indicative of oxidative stress, including elevated measures of reactive oxygen species (ROS) [2] and diminished antioxidant defense, which is associated with lower antioxidant enzymes [3]. Oxidative stress is associated with systemic inflammation, endothelial cell proliferation and apoptosis, and increased vasoconstriction, and thus a noteworthy contributing factor to endothelial dysfunction. In concert, this evidence supports the relationship between oxidative stress, endothelial dysfunction, atherosclerosis, and cardiovascular disease (CVD) [4].

Oxidative stress is a general term for cellular damage caused by an imbalance between pro-oxidants such as ROS and/or reactive nitrogen species (RNS) antioxidants. ROS are oxidizing agents generated during cellular metabolism when the chemical reduction of oxygen forms unstable free radicals, characterized by an unpaired electron [4]. ROS are essential for physiological functions such as gene expression, cellular growth, infection defense, and modulating endothelial function [4–6]. However, to maintain a physiologically beneficial level of ROS within cells, antioxidants are necessary. Antioxidants are enzymatic and nonenzymatic molecules which significantly delay or prevent the oxidizing damage of ROS through the inhibition of ROS formation and action or by repairing cells which have been damaged by ROS [5].

**Fig. 1:**
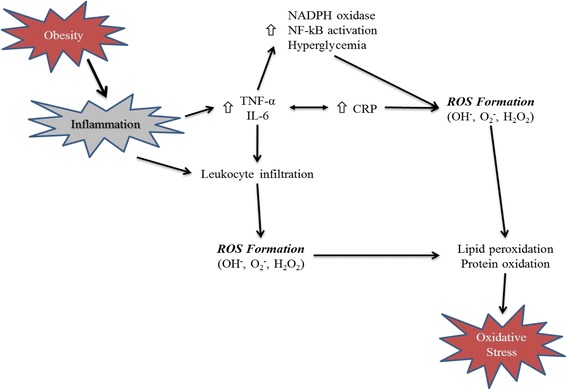
The link between obesity-induced inflammation and oxidative stress. Increased pro-inflammatory response and leukocyte infiltration in obese populations promote the formation of ROS, resulting in oxidative stress. *NADPH* nicotinamide adenine dinucleotide phosphate, *NF-kB* nuclear factor kappa B, *TNF-α* tumor necrosis factor, *IL-6* interleukin-6, *CRP*, C-reactive protein, *OH* ^−^, hydroxyl radical, *O* ^*2*−^ superoxide, *H*_*2*_ *O*_*2*_ hydrogen peroxide, *ROS* reactive oxygen species

Furthermore, obesity-induced inflammation is frequently associated with increased oxidative stress (Fig. 1). Specifically, leptin, an adipocyte-derived hormone, is elevated in obese individuals and can induce oxidative stress [7] and plays a key role in mediating a pro-inflammatory state in obesity [8]; and Korda et al. [7] indicated that this physiological link may help to explain the relationship of obesity, oxidative stress, and inflammation. Additionally, the chronic ingestion of lipid-rich meals can also enhance oxidative stress, lead to weight gain, and facilitate the development of insulin resistance [9]. These negative effects can be attenuated with specific nutrient intake strategies including caloric restriction (CR) and the consumption of exogenous antioxidants. Finally, oxidative stress is elevated during physical activity, but likely serves to instigate a positive antioxidant adaptation [10, 11]. In this review, MEDLINE and PUBMED records were searched using the terms obesity, oxidative stress, inflammation, exercise/physical activity, diets, and antioxidants to identify the studies published in the past 10 years pertaining to two factors that impact obesity-related oxidative stress: physical activity intervention and diet manipulation. Therefore, in this review our aim is to discuss (i) the role of oxidative stress and inflammation as associated with obesity-related diseases, (ii) the potential concerns and benefits of exercise-mediated oxidative stress, and (iii) the advantageous role of dietary modification, including acute or chronic CR and vitamin D supplementation.

### Obesity: a Link between Oxidative Stress and Inflammation

One of the earliest subclinical stages in the atherosclerotic process is an impairment of endothelium-dependent vasodilation, also known as endothelial dysfunction [12]. A mediator of obesity-induced endothelial dysfunction is the level of oxidative stress. Oxidative stress is an imbalance between antioxidants [e.g., superoxide dismutase (SOD) and glutathione peroxidase (GPX)] and reactive oxygen species [e.g., superoxide (O_2_^−^), hydrogen peroxide (H_2_O_2_), and hydroxyl radical (OH^−^)] [13]. Under normal physiological conditions, nitric oxide (NO) is a critical homeostatic regulator of the vessel wall and plays a role in the maintenance of vascular tone and reactivity [14]. However, when ROS production is elevated, the process of cell damage occurs and can possibly facilitate the development of CVD [15] which is largely attributed to oxidation of low-density lipoprotein (LDL) [16]. Several oxidative enzymes such as myeloperoxidase (MPO) and lipoxygenases have been shown to involve in LDL oxidation [17, 18] and are associated with the development of obesity along with inflammation and insulin resistance [19, 20]. Furthermore, increased endogenous activity of antioxidants such as SOD and GPX can decrease the potential for CVD development by regulating ROS and NO production [21, 22]. Importantly, the vascular response to shear stress in obese individuals has been shown to be attenuated [23]. This subsequent attenuation of shear stress has been shown to reduce the activation of endothelial NO synthase (eNOS), resulting in the reduction of NO [24]. The primary sources of ROS in the vasculature are nicotinamide adenine dinucleotide phosphate (NADPH) oxidase, xanthine oxidase, and uncoupled eNOS [25]. Particularly, NADPH oxidase has been found to be the most potent source of O_2_^−^ in the human vasculature [26] and could be activated by LDL and high levels of free fatty acid [27, 28]. Thus, this would help explain the reduced endothelial function in obesity.

More specifically, obese individuals demonstrate elevated markers of ROS, including urinary 8-isoprostanes [2] and decreased antioxidant defenses, represented by lower antioxidant enzymes (e.g., SOD and catalase) [3]. Furthermore, in obese insulin-resistant individuals, the effect of insulin on eNOS is impaired and inducible NO synthase (iNOS) is stimulated, resulting in NO overproduction [29]. Evidence has demonstrated that when expressed iNOS is fully active, it can generate large amount of NO to react with O_2_^−^, resulting in elevated peroxynitrite (ONOO^−^), a powerful reactive oxidant [30]. These findings are further supported by Perticone et al. [31] who demonstrated that abdominal fat distribution and insulin resistance are negatively correlated with forearm blood flow in response to acetylcholine infusion in obese individuals.

Although the mechanisms for obesity-induced oxidative stress remain unclear, leptin, an adipocyte-derived hormone, has been considered as an important contributor. Leptin is responsible for regulating energy intake and expenditure and is also known to play a key role in mediating pro-inflammatory state in obese individuals [8]. Korda et al. [7] have shown that elevated leptin induces oxidative stress (e.g., reduced NO and increased O_2_^−^ and ONOO^−^) in both human endothelial cells and the endothelium of obese mice. Yamagishi et al. [32] have further demonstrated that leptin can increase intracellular ROS generation in microvascular endothelial cells. Thus far, two possible mechanisms have been proposed for the leptin-induced oxidative stress: (i) the stimulation of mitochondrial oxidation of fatty acids [33] and (ii) the elevation of pro-inflammatory cytokines [34].

The pro-inflammatory state of the vessel can negatively impact oxidative stress and play a crucial role in the pathogenesis of obesity-related diseases. Elevated pro-inflammatory cytokines such as tumor necrosis factor (TNF-α) have been shown to downregulate the expression of eNOS (diminishing the dilatory response) in human aortic endothelial cells [34]. Specifically, TNF-α is a potent activator for activation of NADPH oxidase, resulting in the formation of ROS [35]. Picchi et al. [36] also examined the effects of TNF-α administration on oxidative stress response and found that higher O_2_^−^ levels with reduced NO bioavailability in the coronary artery of Zucker obese rats compared to controls.

Interestingly, research has also shown that higher levels of leptin are associated with elevated pro-inflammatory cytokines (e.g., TNF-α and interleukin-6 [IL-6]) [37]. For example, *in vitro* a high dose of leptin has been found to elicit a great amount of TNF-α and IL-6 secretion from activated human peripheral blood mononuclear cells (PBMCs) [38]. In addition, macrophages play a vital role in regulating obese inflammation by the ability to shift T-helper (Th) cell differentiation toward the Th1 subtype, a pro-inflammatory condition [39]. Within the Th1 immune response, the most potent trigger for macrophage-induced ROS production is interferon-gamma (IFN-γ) [40]. Increased ROS such as O_2_^−^, H_2_O_2_, and OH^−^ released by macrophages also provides a positive feedback to upregulate Th1 cell activation [41]. Importantly, leptin has been shown to serve as an immunological adjuvant to efficiently promote Th1 cell response [42]. Thus, inflammation and its subsequent impact on oxidative stress may play a crucial role in the pathogenesis of obesity-related diseases [43, 44].

### Oxidative Stress and Physical Activity

#### Exercise-Induced Oxidative Stress

Exercise-induced oxidative stress has been shown to be dependent upon a number of factors including the mode [the form or type of exercise being utilized (cycling, jogging, and swimming)], intensity [percentage of maximal exercise capacity (VO_2max_)], and duration (total time exercising at a given percentage of VO_2max_) of exercise being performed [45–49]. For example, concentrations of circulating oxidative stress markers were increasingly elevated at greater exercise intensities (25 vs. 50 vs. 75 % VO_2peak_) following 30 min stationary cycling [45, 46] as well as at longer durations (120 vs. 60 vs. 30 min) following stationary cycling at 75 % VO_2peak_ [47]. Additionally, the characteristics of the participant (fitness or training levels, gender, and clinical disease status) can impact the resultant amount of oxidization that occurs [50, 51].

Research has shown that both acute aerobic [52–54] and anaerobic [55–57] exercise can result in increased free radical production, propagating a potential increase in oxidative stress. Further, for oxidative stress to occur, the ROS and RNS produced during exercise must exceed the levels of the antioxidant available for cellular defense, thus resulting in oxidative damage to specific biomolecules [58]. As demonstrated in ironman competitors, the level of antioxidant defenses appear to be adequate to mitigate ROS/RNS production which occurs as a result of high-intensity, but shorter-duration exercise bouts (half ironman competitors), whereas in increasing intensity and/or duration of the acute bout (full ironman competitors), antioxidant defenses can no longer be maintained at sufficient levels, thereby resulting in oxidative damage to surrounding tissues [59]. Both single bouts of aerobic and anaerobic exercises (including resistance-type exercise) can induce oxidative stress, as indicated by the presence of oxidized molecules in a variety of tissue types, especially skeletal muscle [56, 57]. While acute bouts of exercise will lead to associated oxidative stress, these increases seem to be necessary in order to allow for an upregulation in endogenous antioxidant defenses, thus providing beneficial effects to the individual engaged in chronic exercise [60].

Interestingly, the mechanism for increased oxidative stress is somewhat different for aerobic and anaerobic activities. During aerobic exercise, mitochondrial respiration has been purported to produce ROS and RNS; whereas during anaerobic exercise and resistance training, it has been suggested that increases in free radical production may be tempered by enzymatic reactions, prostanoid metabolism, and/or altered calcium homeostasis [48, 49]. It has further been suggested that anaerobically induced oxidative stress may also result from the ischemia/reperfusion cycle of muscle contraction and/or immune system responses following muscle damage that occurs with anaerobic exercise [49, 55, 61]. Similar to aerobic exercise, the results of anaerobic investigations are currently unclear whether the observed increases in ROS and RNS represent a necessary stimulus for adaptation or a detrimental event. While the specific underlying factors that dictate the differences in responses between aerobic and anaerobic physical activities vary, the endpoint of both types of exercise is similar, an elevation in ROS and RNS [48, 62]. An elucidation of the distinct mechanisms may support proposals for different remediation strategies or interventions to limit oxidative stress.

#### Obesity-Related Oxidative Stress

Pro-inflammation has also shown to exert a negative oxidative effect in the skeletal muscle of obese individuals. Obese population exhibit decreased skeletal muscle strength and function compared to healthy weight subjects [63], as well as impaired skeletal muscle mitochondrial respiratory function which contributes to increases in mitochondrial ROS production [64]. Specifically, obese individuals present higher ratios of type II to type I skeletal muscle fibers which have shown to generate two- to threefold more ROS production than type I fibers [64, 65]. Furthermore, Plomgaard et al. [66] demonstrated that TNF-α is solely expressed by type II muscle fibers and serves as a catalyst in skeletal muscle-derived oxidative stress [67]. Additionally, systemic TNF-α administration has been shown to diminish skeletal muscle force production in animal models [68] and directly promotes muscle protein loss [67] via oxidative activation of TNF-α/NF-κB signaling [69]. Interestingly, TNF-α-induced skeletal muscle oxidative stress has been shown to be prevented by antioxidant treatment [67], suggesting that TNF-α may provide a vital target toward correcting obesity-related oxidative stress.

Of special interest is the fact that exercise-induced oxidative stress is exacerbated in obese populations, which has been shown in response to both acute aerobic and resistance exercises. Specifically, the total antioxidant status (TAS) in obese subjects decreased by 8.6 and 17.6 % in response to a single bout of aerobic and resistance exercises, respectively, whereas increases were observed in normal-weight individuals [50]. Furthermore, greater thiobarbituric reactive acid substances (TBARS), a marker of systemic oxidative stress, and lipid hydroperoxide (PEROX) increases were noted in obese subjects [50, 51]. Additionally, despite similar increases in PEROX levels in response to acute aerobic exercise in healthy obese and obese subjects with type 2 diabetes mellitus (T2DM), those with T2DM demonstrated greater decreases in TAS following exercise [70], suggesting a synergistic effect of metabolic dysfunction in further diminishing oxidative stress resistance. The authors suggested these outcomes may be the result of decreased availability of plasma vitamins C and E, elevated systolic blood pressure which may exacerbate vascular production of ROS during exercise, or greater mechanical and metabolic stress imposed by excessive adiposity; however, definitive reasoning remains yet to be elucidated. Finally, a number of assay techniques for the total antioxidant capacity have minimal sensitivity and specificity, thus the results for this measure have limited generalizability.

#### Physical Activity Intervention

Dietary CR as well as aerobic exercise, anaerobic exercise, and resistance training in association with weight loss has been shown to be advantageous in ameliorating oxidative stress and alleviating inflammation in obesity [71–77]. Specifically, overall oxidative stress, as indicated by TBARS and total PEROX, was reduced in healthy obese adults following 24 weeks of resistance-type circuit training [72], potentially due to increases in maximal oxygen consumption and fat-free mass and/or decreases in total fat mass [73]. Additionally, Oh et al. [75] demonstrated that 12 weeks of moderate- to high-intensity aerobic training decreased TBARS and body weight in obese individuals, while baseline levels of the antioxidant GPX were increased following 6 months of aerobic training in obese women [78]. More importantly, following exercise intervention, acute exercise-induced increases in the oxidative stress marker malondialdehyde (MDA) were attenuated while SOD and GPX levels were increased compared to acute exercise-induced responses pre-training [78].

In the absence of weight loss, 3 months of aerobic training in previously sedentary healthy obese adults resulted in significant reductions in skeletal muscle-specific oxidative stress, as indicated by the urinary excretion marker 4-HNE and systemic 8-isoprostane and increased concentrations of mitochondrial antioxidants [79]. Utilizing a similar protocol, Derives et al. [80] reported similar alterations in systemic oxidative stress; however, no change in skeletal muscle 4-HNE expression was observed, suggesting that oxidative stress improvements also occur in other tissue sources. Furthermore, Youssef et al. [81] demonstrated that 12 weeks of moderate aerobic training in the absence of weight loss was also sufficient to attenuate exercise-induced increases of oxidized LDL (ox-LDL) and MPO following an acute bout of maximal aerobic exercise in overweight and obese adolescent girls compared to pre-training responses. Conversely, exercise without weight loss was not sufficient to improve any markers of oxidative stress in obese adolescents as result of 8-week exercise training, despite utilizing higher intensities of exercise [82]. In addition, none of the aforementioned protocols elicited improvements from a pro- to anti-inflammatory state in obese populations at baseline, suggesting that in obese populations: (i) exercise-induced improvements of systemic or skeletal muscle-specific oxidative stress may be the result of intensity and/or duration of the intervention, and (ii) weight loss may be necessary to alter inflammatory profiles.

#### Dietary Intervention

Although exercise training independent of weight loss beneficially increases antioxidant defenses and decreases oxidative stress at baseline and in response to exercise, previous studies suggest at least a 10 % reduction in body weight is necessary to reverse pro-inflammatory parameters which contribute to oxidative stress during obesity [71, 82, 83]. Dramatic weight loss (i.e., gastric bypass surgery) in previously obese men and women has been shown to decreases oxidative stress and vital inflammatory markers, such as IL-6, C-reactive protein, and TNF-α, suggesting that weight loss independently can reduce oxidative stress and inflammation [84, 85]. This strategy may not be feasible or advisable to the general population; however, CR serves as an effective alternative. In animal models, 12 weeks of high-fat feeding increased NADPH oxidase, an important marker in the generation of oxidative stress, and accelerated the pathogenesis of endothelial dysfunction [86]. However, following high-fat diet, rodents underwent an additional 12 weeks of CR with and without exercise training, demonstrating a normalization of NADPH oxidase levels and reversal of the pathological progression of endothelial dysfunction. Furthermore, weight reductions of 10 % following 3 months of dietary restriction (500–1000 kcal/day energy deficit) in obese women resulted in increased glutathione reductase [87], while 6 months of hypocaloric diet elicited weight reductions of nearly 20 % which was sufficient to increase GPX and reduce 8-isoprostane, IL-6, and triglyceride levels in a manner associated with BMI reductions [88]. Additionally, alternate-day dietary restriction (20 %) resulted in a significant reduction in body weight and serum 4-HNE and 8-isoproponate while increasing antioxidant concentrations in obese adults [77]. TNF-α concentrations were also reduced after only 4 weeks, results which persisted throughout the 8-week study [77], potentially contributing to reduced production of cellular ROS [67]. Taken together, research suggests that significant weight fluctuations can directly dictate oxidative stress, inflammatory, and antioxidant enzyme profiles, processes which can be ameliorated through dietary weight lose intervention.

In obese individuals, 6 months of dieting coupled with aerobic exercise training designed to elicit a 10 % reduction in body weight also decreased overall oxidative stress [76]. Interestingly, Roberts et al. [89] reported decreases in oxidative stress accompanied with increases in TAS after only 3 weeks of combined strict dietary intervention and daily aerobic training. Despite no significant reductions in body weight, short-term diet and exercise intervention reduced *in vitro* expression of intracellular adhesion molecule (ICAM), a cellular adhesion marker which serves as an independent marker of CVD and vascular health. Furthermore, monocyte-derived monocyte chemoattractant protein-1 (MCP-1) production was attenuated, results which suggest that macrophage recruitment and exacerbation of the inflammatory response may be improved fairly quickly in response to lifestyle modifications in obese individuals.

Whether aerobic exercise may have a synergistic influence on long-term diet-induced improvements in oxidative stress and inflammatory profiles remains unclear. Wycherley et al. [74] demonstrated that while both diet and diet with aerobic exercise improved oxidative stress and NO availability and significantly reduced body weight, no difference between interventions was observed after 12 weeks in obese individuals with T2DM. Conversely, Ozcelik et al. [90] reported that hypocaloric diet coupled with the weight loss supplement orlistat or aerobic exercise resulted in significant decreases in body weight; however, the diet plus exercise group exhibited significant decreases in oxidative stress while no difference was observed in the diet plus orlistat group.

Both aerobic and anaerobic activities possess the potential to result in increased ROS and RNS production and subsequent oxidative stress. While obesity has been shown to exacerbate the oxidative stress response, dietary manipulation and exercise training may serve as an effective intervention to ameliorate oxidative stress profiles. Whether exercise training improves oxidative stress and inflammatory profiles in the absence of weight loss remains unclear; however, strict CR alone or coupled with physical activity intervention demonstrates promise in alleviating oxidative stress in obese individuals when accompanied with weight reduction.

### Macronutrient-Specific Postprandial Oxidative Stress

Increasing evidence suggests that *postprandial* metabolic responses along with biomarkers of oxidative stress may provide important information regarding an individual’s susceptibility and/or progression of type 2 diabetes as well as other diseases [91, 92]. This is significant since the ingestion of calorie-rich meals may be associated with obesity [93]; however, the ingestion of energy-dense feedings may also elicit potentially deleterious metabolic responses that are independent of chronic weight gain. Several investigations have shown that both the amount [9] and composition [94–96] of macronutrient intake can affect postprandial oxidative stress responses. For example, the ingestion of moderate (75 g) and high (150 g) amounts of dextrose results in minimal oxidative stress as quantified by MDA and H_2_O_2_ [9]. However, the ingestion of 66 g of fat resulted in a significant increase of these two oxidative stress biomarkers [9], which was likely associated with postprandial superoxide production [97]. Varying results were reported following the ingestion of 33 g of fat [98], indicating that not only the source but also the amount of macronutrient distribution can have an effect on postprandial oxidative stress responses. Investigators have also been reported significant increases in postprandial triglycerides (TAG), MDA, H_2_O_2_, and nitrate/nitrite following ingestion of a lipid meal compared to iso-caloric meals of varying macronutrient compositions [94]. Lipid-induced postprandial oxidative stress is likely explained by mitochondrial oxygen leakage and ROS generation [97, 99]. These results suggest potential “stress” associated with the ingestion of lipid-rich meals. It is important to note, however, that these responses can be affected by sex [100, 101]. Goldfarb and colleagues have suggested that this may be related to elevated glutathione status in women compared to men [102].

Interestingly, the magnitude of oxidative stress resulting from the ingestion of a lipid-rich meal has been shown to be greater than that resulting from strenuous exercise [103]. McCarthy et al. [103] was the first to investigate and compare oxidative stress responses from high-fat meals and acute bouts of strenuous exercise. Considering that the subjects in this study were exercise trained, it is possible that a training induced upregulated antioxidant defenses that contributed to the nonsignificant increase in oxidative stress in response to the strenuous exercise [103]. The potentially adverse effects associated with chronic ingestion of lipid-rich meals are apparent (weight gain) and may also be associated with additional ill health, including the development of insulin resistance [9]. Appropriate lifestyle modification (e.g., exercise and/or dietary interventions) seem to diminish oxidative stress and may positively influence diabetes [104] and vascular functioning [105].

### Dietary Modifications and Oxidative Stress

Caloric restriction has been shown to be a successful weight loss intervention that may also improve markers of oxidative stress [106–109]. CR has been shown to promote longevity [110] as well as attenuate morbidity associated with several chronic diseases such as atherosclerosis, diabetes, cancer, autoimmune diseases, renal, neurodegenerative, and respiratory diseases [111]. This dietary modification has been shown to increase lifespan in rodents [112, 113]; however, these findings may not be universal among all animals. Further, a CR diet may be compared to an *ad libitum* diet which may contribute to excessive caloric intake and weight gain [114]. Hence, the validity for CR to promote longevity in humans currently remains a question due to a lack of longitudinal trials. Common approaches to CR include a relative change of macronutrient intake such as decreased carbohydrate or fat intake [115]. Both of which can be coupled with an increase in dietary protein intake. Of these approaches, a relative increase in dietary protein intake may actually contribute to decreased caloric intake since protein of various types (e.g., egg whites, dairy, lean meats) has been shown to have satiating properties [116]. In addition, although dietary fat is also known to induce satiety, higher fat intakes that are commonly coupled with low-carbohydrate diets [115] are potentially dangerous since this can serve as one source of postprandial oxidative stress [9, 94]. CR is usually practiced with a 20–40 % reduction of *ad libitum* dietary intake [117]. This approach has been shown to reduce biomarkers of oxidative stress such as H_2_O_2_, protein carbonyls, and nitrotyrosine as well [106–109].

Two hypotheses have been proposed as potential mechanisms behind the benefits of CR [98]. The hormesis hypothesis suggests that CR acts as a low-intensity stressor and thus, improvements in health and longevity can result as a defense against the exposure [118]. In addition, the oxidative damage hypothesis, which is supported in the literature, suggests that CR itself achieves the same goal by decreasing oxidative stress [119, 120]. Dietary fasting has been shown to prevent atherogenesis by improving NO bioavailability [121–123]. The Daniel Fast [98, 110, 124] is one type of dietary fast involving a plant-based feeding plan that restricts intake of animal products, refined foods, white flour, preservatives, additives, sweeteners, caffeine, and alcohol. Bloomer et al. [98] reported several benefits resulting from 21 days of this *ad libitum* dietary intervention. Improvements of oxidative stress biomarkers and antioxidant status were noted which included a significant reduction in MDA, an increase in nitrate/nitrite, and a 9 % increase in Trolox equivalent antioxidant capacity [98]. Also reported were improvements in blood lipids, glucose, insulin, systolic blood pressure, and body weight [98].

Another dietary modification that may increase antioxidant status, and thus protect against oxidative stress, is the increased consumption of selected micronutrients such as polyphenols [125, 126]. A wealth of data has supported the health benefits associated with increased fruit and vegetable consumption [127–130] which is likely related to the polyphenol antioxidant content [125, 126, 131]. Some classes of polyphenols include anthocyanins, lignans, flavonols, flavanones, flavanol monomers, proanthocyanidins, isoflavones, hydroxycinnamic acids, and hydroxybenzoic acids [126, 132]. Low consumption of fruits and vegetables and excessive fat intake serve as a major risk factor for an unfavorable imbalance between oxidants and antioxidants [9] and the development of chronic diseases contributing to morbidity and mortality [126].

Numerous studies have supported the link between the consumption of isolated antioxidants with benefits including improved antioxidant capacity [133], improved glucose metabolism [134], improved vascular function [135, 136], and attenuated LDL oxidation and progression of atherosclerosis [137]. Examples of these nutraceuticals that have been shown to improve various markers of oxidative stress include (but are not limited to) resveratrol [135, 136, 138, 139], α-lipoic acid [133, 140], ubiquinone (CoQ-10) [141], curcumin [134], quercetin [142], naringin [143], and lycopene [144]. Resveratrol and quercetin have been shown to activate sirtuin-1 (SIRT1) [145, 146]. Resveratrol is found in grapes and red wine [145] and is well known for its anticancer properties [147–149]. Several reports have investigated the SIRT1 activation activity of resveratrol and reported antioxidant, anti-inflammatory, antiapoptotic effects, as well as improvements in vascular functioning [135, 136]. Additional reports demonstrate that the deacetylase activity of SIRT1 is involved in proper glucose metabolism [150], indicating a potential implication for the importance of SIRT1 and related antioxidants for the treatment of insulin resistance [151]. CR has also been shown to upregulate SIRT1 activity [151] as well as increase peroxisome proliferator activated receptor (PPAR)-γ coactivator-1α (PGC-1α) activity, which was associated with improved mitochondrial functioning as well as improvements in oxidative stress, insulin resistance, metabolic rate, and body composition [152]. These studies demonstrate the cellular adaptations that occur in response to CR that can impact oxidative stress.

### The Potential of Vitamin D

Vitamin D insufficiency has shown to correlate with endothelial dysfunction [153], decreased cardiorespiratory fitness [154], and impaired skeletal muscle health, contributing to muscle weakness and decreased function [155]. Furthermore, these conditions may be reversed with vitamin D supplementation [156, 157]. Low vitamin D levels may also serve as a mechanism contributing to the exacerbation of oxidative stress during obesity, a condition worsened by elevated levels of TNF-α [69]; however, research remains limited. *In vivo*, the active vitamin D metabolite, 1,25(OH)_2_D_3_, has been shown to downregulate ICAM-1 expression following peripheral blood mononuclear cell following exposure to TNF-α [158] while serving as an antioxidant at the cellular membrane by decreasing PEROX [159, 160] and increasing TAS as well as oxidative capacity in monocytes [161, 162]. Valcheva et al. [163] recently demonstrated that reactive oxygen species production is enhanced in mice deficient for the vitamin D receptor, while 12 weeks of supplementation decreases circulation markers of oxidative stress as well as improved lipid profiles in type 2 diabetics [164]. In addition, the risk of vitamin D insufficiency is elevated during obesity [165, 166], potentially due to increased sequestering of the steroid in adipose tissue [167]. In fact, for each 1 kg/m^2^ increase in BMI, an estimated decrease of 0.74 nmol/L of vitamin D has been observed [168]. Importantly, vitamin D has been shown to inhibit the production of both TNF-α and IL-6 by downregulating the NF-kB pathway [169, 170].

Tzotzas et al. [171] have provided evidence of the relationship of obesity to vitamin D. In their study, a 10 % reduction in weight resulted in increased vitamin D concentrations in previously vitamin D-insufficient obese individuals [171]. Furthermore, obese individuals supplemented with 3332 IU/day of vitamin D during weight loss intervention resulted in larger decreases in plasma TNF-α and IL-6 compared to placebo [172], while 1000 IU/day of vitamin D coupled with diet and exercise resulted in greater increases in VO_2max_ and weight loss compared to either diet or exercise alone [173]. These results suggest a potential attenuation of oxidative stress in obese individuals supplemented with vitamin D, particularly during exercise. Of note, there are no studies that have investigated the potential role of vitamin D supplementation to suppress oxidative stress in obese individuals during weight loss interventions.

## Conclusions

Aerobic exercise, utilized to reduce obesity, results in an acute state of oxidative stress. However, it is likely that chronic physical activity provides a stimulus for favorable oxidative adaptations and enhanced physiological performance and physical health [13, 73]. Furthermore, while the specific underlying factors that dictate the differences in responses between aerobic and anaerobic exercises vary, the result for both is an elevation in biomarkers of oxidative stress [46, 62]. The mechanisms that would explain the potential benefits from chronic aerobic and anaerobic exercises have not been elucidated. Some have documented that a training-induced increases in endogenous antioxidant status may protect individuals against oxidative stress [78]. Without greater understanding of the distinct mechanisms, it is difficult to propose a specific activity that would result in a specific benefit or outcome.

Numerous studies support the benefits of dietary modification, including vitamin D supplementation, in alleviating oxidative stress; however, the interaction of obesity and physical activity has not been determined. Various metabolic, inflammatory, and cardiovascular mechanisms likely interact to explain the benefits of these interventions. Most importantly, further mechanistic investigations are necessary to determine the most effective intervention(s) for distinct benefits. It does seem evident that weight loss is significant in the alleviation of oxidative stress.

Oxidative stress is strongly associated with obesity, inflammation, vascular function, and diabetes [104, 105]. Appropriate lifestyle modifications can be taken (e.g., exercise training, dietary interventions) to alleviate oxidative stress. A greater understanding of the mechanisms associated with oxidative stress and disease can be utilized in the development of targeted treatment strategies to improve health.

## References

[1] Flegal KM, Carroll MD, Ogden CL, Curtin LR (2010). Prevalence and trends in obesity among US adults, 1999-2008. JAMA.

[2] Keaney JF, Larson MG, Vasan RS, Wilson PW, Lipinska I, Corey D (2003). Obesity and systemic oxidative stress clinical correlates of oxidative stress in the Framingham Study. Arterioscler Thromb Vasc Biol.

[3] Olusi S (2002). Obesity is an independent risk factor for plasma lipid peroxidation and depletion of erythrocyte cytoprotectic enzymes in humans. Int J Obes Relat Metab Disord.

[4] Montezano AC, Touyz RM (2012). Reactive oxygen species and endothelial function–role of nitric oxide synthase uncoupling and Nox family nicotinamide adenine dinucleotide phosphate oxidases. Basic Clin Pharmacol Toxicol.

[5] Kunwar A, Priyadarsini K (2011). Free radicals, oxidative stress and importance of antioxidants in human health. J Med Allied Sci.

[6] Vider J, Laaksonen DE, Kilk A, Atalay M, Lehtmaa J, Zilmer M (2001). Physical exercise induces activation of NF-κB in human peripheral blood lymphocytes. Antioxid Redox Signal.

[7] Korda M, Kubant R, Patton S, Malinski T (2008). Leptin-induced endothelial dysfunction in obesity. Am J Physiol Heart Circ Physiol.

[8] Wannamethee SG, Tchernova J, Whincup P, Lowe G, Kelly A, Rumley A (2007). Plasma leptin: associations with metabolic, inflammatory and haemostatic risk factors for cardiovascular disease. Atherosclerosis.

[9] Bloomer RJ, Kabir MM, Marshall KE, Canale RE, Farney TM (2010). Postprandial oxidative stress in response to dextrose and lipid meals of differing size. Lipids Health Dis.

[10] Miyazaki H, Oh-ishi S, Ookawara T, Kizaki T, Toshinai K, Ha S (2001). Strenuous endurance training in humans reduces oxidative stress following exhausting exercise. Eur J Appl Physiol.

[11] Yavari A, Javadi M, Mirmiran P, Bahadoran Z (2015). Exercise-induced oxidative stress and dietary antioxidants. Asian J Sports Med.

[12] Singhal A (2005). Endothelial dysfunction: role in obesity-related disorders and the early origins of CVD. Proc Nutr Soc.

[13] Sies H (1997). Oxidative stress: oxidants and antioxidants. Exp Physiol.

[14] Verma S, Anderson TJ (2002). Fundamentals of endothelial function for the clinical cardiologist. Circulation.

[15] Ji LL, Gomez-Cabrera MC, Vina J (2006). Exercise and hormesis. Ann N Y Acad Sci.

[16] Zhang P, Xu X, Li X (2014). Cardiovascular diseases: oxidative damage and antioxidant protection. Eur Rev Med Pharmacol Sci.

[17] Carr AC, McCall MR, Frei B (2000). Oxidation of LDL by myeloperoxidase and reactive nitrogen species reaction pathways and antioxidant protection. Arterioscler Thromb Vasc Biol.

[18] Takahashi Y, Zhu H, Yoshimoto T (2005). Essential roles of lipoxygenases in LDL oxidation and development of atherosclerosis. Antioxid Redox Signal.

[19] Wang Q, Xie Z, Zhang W, Zhou J, Wu Y, Zhang M (2014). Myeloperoxidase deletion prevents high-fat diet–induced obesity and insulin resistance. Diabetes.

[20] Neels JG (2013). A role for 5-lipoxygenase products in obesity-associated inflammation and insulin resistance. Adipocyte.

[21] Blankenberg S, Rupprecht HJ, Bickel C, Torzewski M, Hafner G, Tiret L (2003). Glutathione peroxidase 1 activity and cardiovascular events in patients with coronary artery disease. N Engl J Med.

[22] Fukai T, Folz RJ, Landmesser U, Harrison DG (2002). Extracellular superoxide dismutase and cardiovascular disease. Cardiovasc Res.

[23] Arcaro G, Zamboni M, Rossi L, Turcato E, Covi G, Armellini F (1999). Body fat distribution predicts the degree of endothelial dysfunction in uncomplicated obesity. Int J Obes Relat Metab Disord.

[24] Cheang WS, Wong WT, Tian XY, Yang Q, Lee HK, He G-W (2011). Endothelial nitric oxide synthase enhancer reduces oxidative stress and restores endothelial function in db/db mice. Cardiovasc Res.

[25] Hamilton C, Miller W, Al-Benna S, Brosnan M, Drummond R, McBride M (2004). Strategies to reduce oxidative stress in cardiovascular disease. Clin Sci (Lond).

[26] Griendling KK, Ushio-Fukai M (1997). NADH/NADPH oxidase and vascular function. Trends Cardiovasc. Med..

[27] Berry C, Hamilton CA, Brosnan MJ, Magill FG, Berg GA, McMurray JJ (2000). Investigation into the sources of superoxide in human blood vessels angiotensin II increases superoxide production in human internal mammary arteries. Circulation.

[28] Inoguchi T, Li P, Umeda F, Yu HY, Kakimoto M, Imamura M (2000). High glucose level and free fatty acid stimulate reactive oxygen species production through protein kinase C-dependent activation of NAD(P)H oxidase in cultured vascular cells. Diabetes.

[29] Dallaire P, Marette A (2004). Obesity-linked insulin resistance: is nitric oxide the missing link. Can J Diabetes.

[30] Pacher P, Beckman JS, Liaudet L (2007). Nitric oxide and peroxynitrite in health and disease. Physiol Rev.

[31] Perticone F, Ceravolo R, Candigliota M, Ventura G, Iacopino S, Sinopoli F (2001). Obesity and body fat distribution induce endothelial dysfunction by oxidative stress protective effect of vitamin C. Diabetes.

[32] S-i Y, Amano S, Inagaki Y, Okamoto T, Takeuchi M, Inoue H (2003). Pigment epithelium-derived factor inhibits leptin-induced angiogenesis by suppressing vascular endothelial growth factor gene expression through anti-oxidative properties. Microvasc Res.

[33] S-i Y, Edelstein D, Du X-l, Kaneda Y, Guzmán M, Brownlee M (2001). Leptin induces mitochondrial superoxide production and monocyte chemoattractant protein-1 expression in aortic endothelial cells by increasing fatty acid oxidation via protein kinase A. J Biol Chem.

[34] Zhang H, Park Y, Wu J, Chen X, Lee S, Yang J (2009). Role of TNF-alpha in vascular dysfunction. Clin Sci (Lond).

[35] Yan S, Zhang X, Zheng H, Hu D, Zhang Y, Guan Q (2014). Clematichinenoside inhibits VCAM-1 and ICAM-1 expression in TNF-α-treated endothelial cells via NADPH oxidase-dependent IκB kinase/NF-κB pathway. Free Radic Biol Med.

[36] Picchi A, Gao X, Belmadani S, Potter BJ, Focardi M, Chilian WM (2006). Tumor necrosis factor-α induces endothelial dysfunction in the prediabetic metabolic syndrome. Circ Res.

[37] Zumbach MS, Boehme MWJ, Wahl P, Stremmel W, Ziegler R, Nawroth PP (1997). Tumor necrosis factor increases serum leptin levels in humans. J Clin Endocrinol Metab.

[38] Zarkesh-Esfahani H, Pockley G, Metcalfe RA, Bidlingmaier M, Wu Z, Ajami A (2001). High-dose leptin activates human leukocytes via receptor expression on monocytes. J Immunol.

[39] Lumeng CN, Saltiel AR (2011). Inflammatory links between obesity and metabolic disease. J Clin Invest.

[40] Nathan CF, Murray HW, Wiebe M, Rubin BY (1983). Identification of interferon-gamma as the lymphokine that activates human macrophage oxidative metabolism and antimicrobial activity. J Exp Med.

[41] Murr C, Schroecksnadel K, Winkler C, Ledochowski M, Fuchs D (2005). Antioxidants may increase the probability of developing allergic diseases and asthma. Med Hypotheses.

[42] Cojocaru M, Cojocaru IM, Silosi I, Rogoz S (2013). Role of leptin in autoimmune diseases. Maedica (Buchar).

[43] Maingrette F, Renier G (2003). Leptin increases lipoprotein lipase secretion by macrophages: involvement of oxidative stress and protein kinase C. Diabetes.

[44] Youn J-Y, Siu KL, Lob HE, Itani H, Harrison DG, Cai H (2014). Role of vascular oxidative stress in obesity and metabolic syndrome. Diabetes.

[45] Bloomer RJ, Goldfarb AH, Wideman L, McKenzie MJ, Consitt LA (2005). Effects of acute aerobic and anaerobic exercise on blood markers of oxidative stress. J Strength Cond Res.

[46] Bloomer RJ, Goldfarb AH (2004). Anaerobic exercise and oxidative stress: a review. Can J Appl Physiol.

[47] Goto C, Higashi Y, Kimura M, Noma K, Hara K, Nakagawa K (2003). Effect of different intensities of exercise on endothelium-dependent vasodilation in humans: role of endothelium-dependent nitric oxide and oxidative stress. Circulation.

[48] Goto C, Nishioka K, Umemura T, Jitsuiki D, Sakagutchi A, Kawamura M (2007). Acute moderate-intensity exercise induces vasodilation through an increase in nitric oxide bioavailability in humans. Am J Hypertens.

[49] Bloomer R, Davis P, Consitt L, Wideman L (2007). Plasma protein carbonyl response to increasing exercise duration in aerobically trained men and women. Int J Sports Med.

[50] Vincent HK, Morgan JW, Vincent KR (2004). Obesity exacerbates oxidative stress levels after acute exercise. Med Sci Sports Exerc.

[51] Vincent HK, Vincent KR, Bourguignon C, Braith RW (2005). Obesity and postexercise oxidative stress in older women. Med Sci Sports Exerc.

[52] Davies KJ, Quintanilha AT, Brooks GA, Packer L (1982). Free radicals and tissue damage produced by exercise. Biochem Biophys Res Commun.

[53] Ashton T, Rowlands CC, Jones E, Young IS, Jackson SK, Davies B (1998). Electron spin resonance spectroscopic detection of oxygen-centred radicals in human serum following exhaustive exercise. Eur J Appl Physiol Occup Physiol.

[54] Ashton T, Young IS, Peters JR, Jones E, Jackson SK, Davies B (1999). Electron spin resonance spectroscopy, exercise, and oxidative stress: an ascorbic acid intervention study. J Appl Physiol.

[55] Groussard C, Rannou-Bekono F, Machefer G, Chevanne M, Vincent S, Sergent O (2003). Changes in blood lipid peroxidation markers and antioxidants after a single sprint anaerobic exercise. Eur J Appl Physiol.

[56] Bailey DM, Lawrenson L, Mceneny J, Young IS, James PE, Jackson SK (2007). Electron paramagnetic spectroscopic evidence of exercise-induced free radical accumulation in human skeletal muscle. Free Radic Res.

[57] Bailey DM, Young IS, McEneny J, Lawrenson L, Kim J, Barden J (2004). Regulation of free radical outflow from an isolated muscle bed in exercising humans. Am J Physiol Heart Circ Physiol.

[58] Halliwell B (1984). Oxygen radicals: a commonsense look at their nature and medical importance. Med Biol.

[59] Knez WL, Jenkins DG, Coombes JS (2007). Oxidative stress in half and full Ironman triathletes. Med Sci Sports Exerc.

[60] Fisher-Wellman K, Bloomer RJ (2009). Acute exercise and oxidative stress: a 30 year history. Dyn Med.

[61] McAnulty SR, McAnulty LS, Nieman DC, Morrow JD, Utter AC, Dumke CL (2005). Effect of resistance exercise and carbohydrate ingestion on oxidative stress. Free Radic Res.

[62] Hartmann A, Niess AM, Sen C, Packer L, Hänninen O (2000). Oxidative DNA damage in exercise. Handbook of oxidants and antioxidants in exercise.

[63] Hilton TN, Tuttle LJ, Bohnert KL, Mueller MJ, Sinacore DR (2008). Excessive adipose tissue infiltration in skeletal muscle in individuals with obesity, diabetes mellitus, and peripheral neuropathy: association with performance and function. Phys Ther.

[64] Hey-Mogensen M, Højlund K, Vind B, Wang L, Dela F, Beck-Nielsen H (2010). Effect of physical training on mitochondrial respiration and reactive oxygen species release in skeletal muscle in patients with obesity and type 2 diabetes. Diabetologia.

[65] Anderson EJ, Neufer PD (2006). Type II skeletal myofibers possess unique properties that potentiate mitochondrial H_2_O_2_ generation. Am J Physiol Cell Physiol.

[66] Plomgaard P, Penkowa M, Pedersen BK (2005). Fiber type specific expression of TNF-alpha, IL-6 and IL-18 in human skeletal muscles. Exerc Immunol Rev.

[67] Li Y-P, Reid MB (2000). NF-κB mediates the protein loss induced by TNF-α in differentiated skeletal muscle myotubes. Am J Physiol Regul Integr Comp Physiol.

[68] Wilcox P, Wakai Y, Walley K, Cooper D, Road J (1994). Tumor necrosis factor alpha decreases in vivo diaphragm contractility in dogs. Am J Respir Crit Care Med.

[69] Reid M, Li YP (2001). Cytokines and oxidative signalling in skeletal muscle. Acta Physiol Scand.

[70] Brinkmann C, Chung N, Schmidt U, Kreutz T, Lenzen E, Schiffer T (2012). Training alters the skeletal muscle antioxidative capacity in non‐insulin‐dependent type 2 diabetic men. Scand J Med Sci Sports.

[71] Bruun JM, Helge JW, Richelsen B, Stallknecht B (2005). Diet and exercise reduce low-grade inflammation and macrophage infiltration in adipose tissue but not in skeletal muscle in severely obese subjects. Am J Physiol Endocrinol Metab.

[72] Phillips MD, Patrizi RM, Cheek DJ, Wooten JS, Barbee JJ, Mitchell JB (2012). Resistance training reduces subclinical inflammation in obese, postmenopausal women. Med Sci Sports Exerc.

[73] Vincent HK, Bourguignon C, Vincent KR (2006). Resistance training lowers exercise‐induced oxidative stress and homocysteine levels in overweight and obese older adults. Obesity.

[74] Wycherley TP, Brinkworth GD, Noakes M, Buckley JD, Clifton PM (2008). Effect of caloric restriction with and without exercise training on oxidative stress and endothelial function in obese subjects with type 2 diabetes. Diabetes Obes Metab.

[75] Oh S, Tanaka K, Warabi E, Shoda J (2013). Exercise reduces inflammation and oxidative stress in obesity-related liver diseases. Med Sci Sports Exerc.

[76] Rector RS, Warner SO, Liu Y, Hinton PS, Sun GY, Cox RH (2007). Exercise and diet induced weight loss improves measures of oxidative stress and insulin sensitivity in adults with characteristics of the metabolic syndrome. Am J Physiol Endocrinol Metab.

[77] Johnson JB, Summer W, Cutler RG, Martin B, Hyun D-H, Dixit VD (2007). Alternate day calorie restriction improves clinical findings and reduces markers of oxidative stress and inflammation in overweight adults with moderate asthma. Free Radic Biol Med.

[78] Shin Y-A, Lee J-H, Song W, Jun T-W (2008). Exercise training improves the antioxidant enzyme activity with no changes of telomere length. Mech Ageing Dev.

[79] Samjoo I, Safdar A, Hamadeh M, Raha S, Tarnopolsky M (2013). The effect of endurance exercise on both skeletal muscle and systemic oxidative stress in previously sedentary obese men. Nutr Diabetes.

[80] Devries MC, Hamadeh MJ, Glover AW, Raha S, Samjoo IA, Tarnopolsky MA (2008). Endurance training without weight loss lowers systemic, but not muscle, oxidative stress with no effect on inflammation in lean and obese women. Free Radic Biol Med.

[81] Youssef H, Groussard C, Lemoine-Morel S, Pincemail J, Jacob C, Moussa E (2015). Aerobic training suppresses exercise-induced lipid peroxidation and inflammation in overweight/obese adolescent girls. Pediatr Exerc Sci.

[82] Kelly AS, Steinberger J, Olson TP, Dengel DR (2007). In the absence of weight loss, exercise training does not improve adipokines or oxidative stress in overweight children. Metabolism.

[83] Forsythe LK, Wallace JM, Livingstone MBE (2008). Obesity and inflammation: the effects of weight loss. Nutr Res Rev.

[84] Kopp H-P, Kopp C, Festa A, Krzyzanowska K, Kriwanek S, Minar E (2003). Impact of weight loss on inflammatory proteins and their association with the insulin resistance syndrome in morbidly obese patients. Arterioscler Thromb Vasc Biol.

[85] Gletsu‐Miller N, Hansen JM, Jones DP, Go YM, Torres WE, Ziegler TR (2009). Loss of total and visceral adipose tissue mass predicts decreases in oxidative stress after weight‐loss surgery. Obesity.

[86] Touati S, Montezano AC, Meziri F, Riva C, Touyz RM, Laurant P (2015). Exercise training protects against atherosclerotic risk factors through vascular NADPH oxidase, ERK1/2 and SAPK/JNK down‐regulation in obese rats. Clin Exp Pharmacol Physiol.

[87] Ramezanipour M, Jalali M, Sadrzade-Yeganeh H, Keshavarz SA, Eshraghian MR, Bagheri M (2014). The effect of weight reduction on antioxidant enzymes and their association with dietary intake of vitamins A, C and E. Arq Bras Endocrinol Metabol.

[88] Bougoulia M, Triantos A, Koliakos G (2006). Plasma interleukin-6 levels, glutathione peroxidase and isoprostane in obese women before and after weight loss. Association with cardiovascular risk factors. Hormones (Athens).

[89] Roberts CK, Won D, Pruthi S, Kurtovic S, Sindhu RK, Vaziri ND (2006). Effect of a short-term diet and exercise intervention on oxidative stress, inflammation, MMP-9, and monocyte chemotactic activity in men with metabolic syndrome factors. J Appl Physiol.

[90] Ozcelik O, Ozkan Y, Karatas F, Kelestimur H (2005). Exercise training as an adjunct to orlistat therapy reduces oxidative stress in obese subjects. Tohoku J Exp Med.

[91] O’Keefe JH, Bell DS (2007). Postprandial hyperglycemia/hyperlipidemia (postprandial dysmetabolism) is a cardiovascular risk factor. Am J Crdiol.

[92] Pastromas S, Terzi A-B, Tousoulis D, Koulouris S (2008). Postprandial lipemia: an under-recognized atherogenic factor in patients with diabetes mellitus. Int J Cardiol.

[93] Rosenheck R (2008). Fast food consumption and increased caloric intake: a systematic review of a trajectory towards weight gain and obesity risk. Obes Rev.

[94] Fisher-Wellman KH, Bloomer RJ (2010). Exacerbated postprandial oxidative stress induced by the acute intake of a lipid meal compared to isoenergetically administered carbohydrate, protein, and mixed meals in young, healthy men. J Am Coll Nutr.

[95] Rebolledo O, Dato SA (2005). Postprandial hyperglycemia and hyperlipidemia-generated glycoxidative stress: its contribution to the pathogenesis of diabetes complications. Eur Rev Med Pharmacol Sci.

[96] Sies H, Stahl W, Sevanian A (2005). Nutritional, dietary and postprandial oxidative stress. J Nutr.

[97] Liu Y, Fiskum G, Schubert D (2002). Generation of reactive oxygen species by the mitochondrial electron transport chain. J Neurochem.

[98] Bloomer RJ, Kabir MM, Trepanowski JF, Canale RE, Farney TM (2011). A 21 day Daniel Fast improves selected biomarkers of antioxidant status and oxidative stress in men and women. Nutr Metab (Lond).

[99] Ruggiero C, Ehrenshaft M, Cleland E, Stadler K (2011). High-fat diet induces an initial adaptation of mitochondrial bioenergetics in the kidney despite evident oxidative stress and mitochondrial ROS production. Am J Physiol Endocrinol Metab.

[100] Bloomer RJ, Ferebee DE, Fisher-Wellman KH, Quindry JC, Schilling BK (2009). Postprandial oxidative stress: influence of sex and exercise training status. Med Sci Sports Exerc.

[101] Bloomer RJ, Fisher-Wellman KH (2010). Lower postprandial oxidative stress in women compared with men. Gend Med.

[102] Goldfarb AH, McKenzie MJ, Bloomer RJ (2007). Gender comparisons of exercise-induced oxidative stress: influence of antioxidant supplementation. Appl Physiol Nutr Metab.

[103] McCarthy CG, Farney TM, Canale RE, Dessoulavy ME, Bloomer RJ (2013). High-fat feeding, but not strenuous exercise, increases blood oxidative stress in trained men. Appl Physiol Nutr Metab.

[104] Giacco F, Brownlee M (2010). Oxidative stress and diabetic complications. Circ Res.

[105] Ramadan R, Dhawan SS, Syed H, Pohlel FK, Binongo JN, Ghazzal ZB (2014). Effects of clopidogrel therapy on oxidative stress, inflammation, vascular function, and progenitor cells in stable coronary artery disease. J Cardiovasc Pharmacol.

[106] Bevilacqua L, Ramsey JJ, Hagopian K, Weindruch R, Harper M-E (2004). Effects of short-and medium-term calorie restriction on muscle mitochondrial proton leak and reactive oxygen species production. Am J Physiol Endocrinol Metab.

[107] Hagopian K, Harper M-E, Ram JJ, Humble SJ, Weindruch R, Ramsey JJ (2005). Long-term calorie restriction reduces proton leak and hydrogen peroxide production in liver mitochondria. Am J Physiol Endocrinol Metab.

[108] Hyun D-H, Emerson SS, Jo D-G, Mattson MP, de Cabo R (2006). Calorie restriction up-regulates the plasma membrane redox system in brain cells and suppresses oxidative stress during aging. Proc Natl Acad Sci U S A.

[109] Zainal TA, Oberley TD, Allison DB, Szweda LI, Weindruch R (2000). Caloric restriction of rhesus monkeys lowers oxidative damage in skeletal muscle. FASEB J.

[110] Trepanowski JF, Canale RE, Marshall KE, Kabir MM, Bloomer RJ (2011). Impact of caloric and dietary restriction regimens on markers of health and longevity in humans and animals: a summary of available findings. Nutr J.

[111] S-i I (2009). SIRT1 and caloric restriction: an insight into possible trade-offs between robustness and frailty. Curr Opin Clin Nutr Metab Care.

[112] Goldberg EL, Romero‐Aleshire MJ, Renkema KR, Ventevogel MS, Chew WM, Uhrlaub JL (2015). Lifespan‐extending caloric restriction or mTOR inhibition impair adaptive immunity of old mice by distinct mechanisms. Aging Cell.

[113] Jové M, Naudí A, Ramírez‐Núñez O, Portero‐Otín M, Selman C, Withers DJ (2014). Caloric restriction reveals a metabolomic and lipidomic signature in liver of male mice. Aging cell.

[114] Sohal RS, Forster MJ (2014). Caloric restriction and the aging process: a critique. Free Radic Biol Med.

[115] Noakes TD (2013). Low-carbohydrate and high-fat intake can manage obesity and associated conditions: occasional survey. S Afr Med J.

[116] Kremsdorf RA, Hoofnagle AN, Kratz M, Weigle DS, Callahan HS, Purnell JQ (2013). Effects of a high-protein diet on regulation of phosphorus homeostasis. J Clin Endocrinol Metab.

[117] Cantó C, Auwerx J (2009). Caloric restriction, SIRT1 and longevity. Trends Endocrinol Metab.

[118] Masoro EJ (2000). Caloric restriction and aging: an update. Exp Gerontol.

[119] Gredilla R, Barja G (2005). Minireview: the role of oxidative stress in relation to caloric restriction and longevity. Endocrinology.

[120] Sohal RS, Weindruch R (1996). Oxidative stress, caloric restriction, and aging. Science.

[121] Nisoli E, Tonello C, Cardile A, Cozzi V, Bracale R, Tedesco L (2005). Calorie restriction promotes mitochondrial biogenesis by inducing the expression of eNOS. Science.

[122] Kondo M, Shibata R, Miura R, Shimano M, Kondo K, Li P (2009). Caloric restriction stimulates revascularization in response to ischemia via adiponectin-mediated activation of endothelial nitric-oxide synthase. J Biol Chem.

[123] Monda M, Messina G, Scognamiglio I, Lombardi A, Martin GA, Sperlongano P (2014). Short-term diet and moderate exercise in young overweight men modulate cardiocyte and hepatocarcinoma survival by oxidative stress. Oxid Med Cell Longev.

[124] Trepanowski JF, Kabir MM, Alleman RJ, Bloomer RJ (2012). A 21-day Daniel fast with or without krill oil supplementation improves anthropometric parameters and the cardiometabolic profile in men and women. Nutr Metab (Lond).

[125] Mennen LI, Sapinho D, de Bree A, Arnault N, Bertrais S, Galan P (2004). Consumption of foods rich in flavonoids is related to a decreased cardiovascular risk in apparently healthy French women. J Nutr.

[126] Urquiaga I, Leighton F (2000). Plant polyphenol antioxidants and oxidative stress. Biol Res.

[127] Liu RH (2003). Health benefits of fruit and vegetables are from additive and synergistic combinations of phytochemicals. Am J Clin Nutr.

[128] Lotito SB, Frei B (2006). Consumption of flavonoid-rich foods and increased plasma antioxidant capacity in humans: cause, consequence, or epiphenomenon?. Free Radic Biol Med.

[129] Tuso P, Stoll SR, Li WW (2015). A plant-based diet, atherogenesis, and coronary artery disease prevention. Perm J.

[130] Jiang Y, Wu S-H, Shu X-O, Xiang Y-B, Ji B-T, Milne GL (2014). Cruciferous vegetable intake is inversely correlated with circulating levels of proinflammatory markers in women. J Acad Nutr Diet.

[131] Feskanich D, Ziegler RG, Michaud DS, Giovannucci EL, Speizer FE, Willett WC (2000). Prospective study of fruit and vegetable consumption and risk of lung cancer among men and women. J Natl Cancer Inst.

[132] Manach C, Williamson G, Morand C, Scalbert A, Rémésy C (2005). Bioavailability and bioefficacy of polyphenols in humans. I. Review of 97 bioavailability studies. Am J Clin Nutr.

[133] Nickander KK, Mcphee BR, Low PA, Tritschler H (1996). Alpha-lipoic acid: antioxidant potency against lipid peroxidation of neural tissues in vitro and implications for diabetic neuropathy. Free Radic Biol Med.

[134] Zhang D-W, Fu M, Gao S-H, Liu J-L (2013). Curcumin and diabetes: a systematic review. Evid Based Complement Alternat Med.

[135] Csiszar A, Labinskyy N, Podlutsky A, Kaminski PM, Wolin MS, Zhang C (2008). Vasoprotective effects of resveratrol and SIRT1: attenuation of cigarette smoke-induced oxidative stress and proinflammatory phenotypic alterations. Am J Physiol Heart Circ Physiol.

[136] Ungvari Z, Orosz Z, Rivera A, Labinskyy N, Xiangmin Z, Olson S (2007). Resveratrol increases vascular oxidative stress resistance. Am J Physiol Heart Circ Physiol.

[137] Kumar A, Kaur H, Devi P, Mohan V (2009). Role of coenzyme Q10 (CoQ10) in cardiac disease, hypertension and Meniere-like syndrome. Pharmacol Ther.

[138] Kasdallah-Grissa A, Mornagui B, Aouani E, Hammami M, El May M, Gharbi N (2007). Resveratrol, a red wine polyphenol, attenuates ethanol-induced oxidative stress in rat liver. Life Sci.

[139] Kode A, Rajendrasozhan S, Caito S, Yang S-R, Megson IL, Rahman I (2008). Resveratrol induces glutathione synthesis by activation of Nrf2 and protects against cigarette smoke-mediated oxidative stress in human lung epithelial cells. Am J Physiol Lung Cell Mol Physiol.

[140] Stanković MN, Mladenović D, Ninković M, Ðuričić I, Šobajić S, Jorgačević B (2014). The effects of α-lipoic acid on liver oxidative stress and free fatty acid composition in methionine–choline deficient diet-induced NAFLD. J Med Food.

[141] Tarry-Adkins JL, Blackmore HL, Martin-Gronert MS, Fernandez-Twinn DS, McConnell JM, Hargreaves IP (2013). Coenzyme Q10 prevents accelerated cardiac aging in a rat model of poor maternal nutrition and accelerated postnatal growth. Mol Metab.

[142] Wang L, Cheng X, Li H, Qiu F, Yang N, Wang B (2014). Quercetin reduces oxidative stress and inhibits activation of c-Jun N-terminal kinase/activator protein-1 signaling in an experimental mouse model of abdominal aortic aneurysm. Mol Med Rep.

[143] Chen J, Guo R, Yan H, Tian L, You Q, Li S (2014). Naringin inhibits ROS‐activated MAPK pathway in high glucose‐induced injuries in H9c2 cardiac cells. Basic Clin Pharmacol Toxicol.

[144] Fletcher NM, Awonuga AO, Saed MG, Abu-Soud HM, Diamond MP, Saed GM (2013). Lycopene, a powerful antioxidant, significantly reduces the development of the adhesion phenotype. Syst Biol Reprod Med.

[145] Borra MT, Smith BC, Denu JM (2005). Mechanism of human SIRT1 activation by resveratrol. J Biol Chem.

[146] Lagouge M, Argmann C, Gerhart-Hines Z, Meziane H, Lerin C, Daussin F (2006). Resveratrol improves mitochondrial function and protects against metabolic disease by activating SIRT1 and PGC-1α. Cell.

[147] Amiri F, Zarnani A-H, Zand H, Koohdani F, Jeddi-Tehrani M, Vafa M (2013). Synergistic anti-proliferative effect of resveratrol and etoposide on human hepatocellular and colon cancer cell lines. Eur J Pharmacol.

[148] Ulasli SS, Celik S, Gunay E, Ozdemir M, Hazman O, Ozyurek A (2013). Anticancer effects of thymoquinone, caffeic acid phenethyl ester and resveratrol on A549 non-small cell lung cancer cells exposed to benzo(a)pyrene. Asian Pac J Cancer Prev.

[149] Jung K-H, Lee JH, Park JW, Quach CHT, Moon S-H, Cho YS (2014). Resveratrol-loaded polymeric nanoparticles suppress glucose metabolism and tumor growth in vitro and in vivo. Int J Pharm.

[150] Caron AZ, He X, Mottawea W, Seifert EL, Jardine K, Dewar-Darch D (2014). The SIRT1 deacetylase protects mice against the symptoms of metabolic syndrome. FASEB J.

[151] Kitada M, Koya D (2013). SIRT1 in type 2 diabetes: mechanisms and therapeutic potential. Diabetes Metab J.

[152] Civitarese AE, Carling S, Heilbronn LK, Hulver MH, Ukropcova B, Deutsch WA (2007). Calorie restriction increases muscle mitochondrial biogenesis in healthy humans. PLoS Med.

[153] Nemerovski CW, Dorsch MP, Simpson RU, Bone HG, Aaronson KD, Bleske BE (2009). Vitamin D and cardiovascular disease. Pharmacotherapy.

[154] Mowry DA, Costello MM, Heelan KA (2009). Association among cardiorespiratory fitness, body fat, and bone marker measurements in healthy young females. J Am Osteopath Assoc.

[155] Moran DS, McClung JP, Kohen T, Lieberman HR (2013). Vitamin D and physical performance. Sports Med.

[156] Cannell JJ, Hollis BW, Sorenson MB, Taft TN, Anderson J (2009). Athletic performance and vitamin D. Med Sci Sports Exerc.

[157] Sato Y, Iwamoto J, Kanoko T, Satoh K (2005). Low-dose vitamin D prevents muscular atrophy and reduces falls and hip fractures in women after stroke: a randomized controlled trial. Cerebrovasc Dis.

[158] Martinesi M, Treves C, d'Albasio G, Bagnoli S, Bonanomi AG, Stio M (2008). Vitamin D derivatives induce apoptosis and downregulate ICAM-1 levels in peripheral blood mononuclear cells of inflammatory bowel disease patients. Inflamm Bowel Dis.

[159] Garcion E, Sindji L, Leblondel G, Brachet P, Darcy F (1999). 1, 25‐Dihydroxyvitamin D3 regulates the synthesis of γ‐glutamyl transpeptidase and glutathione levels in rat primary astrocytes. J Neurochem.

[160] Wiseman H (1993). Vitamin D, is a membrane antioxidant ability to inhibit iron-dependent lipid peroxidation in liposomes compared to cholesterol, ergosterol and tamoxifen and relevance to anticancer action. FEBS Lett.

[161] Cohen MS, Mesler DE, Snipes RG, Gray T (1986). 1, 25-Dihydroxyvitamin D3 activates secretion of hydrogen peroxide by human monocytes. J Immunol.

[162] Wu C-C, Chang J-H, Chen C-C, Su S-B, Yang L-K, Ma W-Y (2011). Calcitriol treatment attenuates inflammation and oxidative stress in hemodialysis patients with secondary hyperparathyroidism. Tohoku J Exp Med.

[163] Valcheva P, Cardus A, Panizo S, Parisi E, Bozic M, Lopez Novoa JM (2014). Lack of vitamin D receptor causes stress-induced premature senescence in vascular smooth muscle cells through enhanced local angiotensin-II signals. Atherosclerosis.

[164] Eftekhari MH, Akbarzadeh M, Dabbaghmanesh MH, Hassanzadeh J (2014). The effect of calcitriol on lipid profile and oxidative stress in hyperlipidemic patients with type 2 diabetes mellitus. ARYA Atheroscler.

[165] Konradsen S, Ag H, Lindberg F, Hexeberg S, Jorde R (2008). Serum 1, 25-dihydroxy vitamin D is inversely associated with body mass index. Eur J Nutr.

[166] Parikh SJ, Edelman M, Uwaifo GI, Freedman RJ, Semega-Janneh M, Reynolds J (2004). The relationship between obesity and serum 1, 25-dihydroxy vitamin D concentrations in healthy adults. J Clin Endocrinol Metab.

[167] Wortsman J, Matsuoka LY, Chen TC, Lu Z, Holick MF (2007). Decreased bioavailability of vitamin D in obesity. Am J Clin Nutr.

[168] McGill A-T, Stewart JM, Lithander FE, Strik CM, Poppitt SD (2008). Relationships of low serum vitamin D3 with anthropometry and markers of the metabolic syndrome and diabetes in overweight and obesity. Nutr J.

[169] Khoo A-L, Chai LY, Koenen HJ, Kullberg B-J, Joosten I, van der Ven AJ (2011). 1, 25-Dihydroxyvitamin D3 modulates cytokine production induced by Candida albicans: impact of seasonal variation of immune responses. J Infect Dis.

[170] Li B, Baylink DJ, Deb C, Zannetti C, Rajaallah F, Xing W (2013). 1, 25-Dihydroxyvitamin D3 suppresses TLR8 expression and TLR8-mediated inflammatory responses in monocytes in vitro and experimental autoimmune encephalomyelitis in vivo. PLoS One.

[171] Tzotzas T, Papadopoulou FG, Tziomalos K, Karras S, Gastaris K, Perros P (2010). Rising serum 25-hydroxy-vitamin D levels after weight loss in obese women correlate with improvement in insulin resistance. J Clin Endocrinol Metab.

[172] Zittermann A, Frisch S, Berthold HK, Götting C, Kuhn J, Kleesiek K (2009). Vitamin D supplementation enhances the beneficial effects of weight loss on cardiovascular disease risk markers. Am J Clin Nutr.

[173] Villareal DT, Chode S, Parimi N, Sinacore DR, Hilton T, Armamento-Villareal R (2011). Weight loss, exercise, or both and physical function in obese older adults. N Engl J Med.

